# ZnX_2_ mediated post-synthetic transformation of zero dimensional Cs_4_PbBr_6_ nanocrystals for opto-electronic applications†

**DOI:** 10.1039/c9na00244h

**Published:** 2019-05-08

**Authors:** Sumit Kumar Sharma, Swati Mamgain, Burhanuddin Attarwala, Aswani Yella

**Affiliations:** Centre for Research in Nanotechnology and Science, Indian Institute of Technology Bombay 400076 India aswani.yella@iitb.ac.in; Department of Metallurgical Engineering and Materials Science, Indian Institute of Technology Bombay 400076 India

## Abstract

Herein we demonstrate a facile approach for the synthesis of all inorganic cesium lead halide perovskite nanocrystal composites CsPbX_3_ (X = Cl, Br, I) with high quantum yield by post-synthetic modulation of zero dimensional Cs_4_PbBr_6_ nanocrystals with ZnX_2_ salts. The transformation of Cs_4_PbBr_6_ nanocrystals into CsPbBr_3_ takes place in two steps, the first step being the surface modification of the Cs_4_PbBr_6_ nanocrystals with Zn^2+^ ions and the second step being extraction of CsBr by the Zn^2+^ ions resulting in the formation of composite Cs_4_PbBr_6_/CsPbBr_3_ nanocrystals. The transformed composite nanocrystals were found to have a PL QY exceeding 90% and the shape of the nanocrystals also changed from hexagonal to cubic shaped. Owing to the highly ionic nature of the nanocrystals, complete anion exchange could be also realized using ZnI_2_ salt. In the case of the iodide post-treated samples, nanorods were obtained which exhibited bright red photoluminescence. Photodetectors based on the ZnI_2_ treated Cs_4_PbBr_6_ NCs were fabricated, and the photodetectors exhibited a high on/off ratio with a fast response time. The excellent optoelectronic properties make this treatment versatile for a wide range of functional optoelectronic devices like light emitting diodes and photovoltaic devices.

## Abstract 2

Herein we demonstrate a facile approach for the synthesis of all inorganic cesium lead halide perovskite nanocrystal composites CsPbX_3_ (X = Cl, Br, I) with high quantum yield by post-synthetic modulation of zero dimensional Cs_4_PbBr_6_ nanocrystals with ZnX_2_ salts.

## Graphical abstract

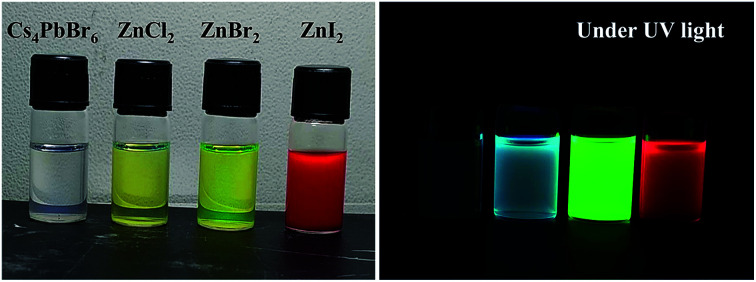

## Introduction

Lead halide perovskite NCs (especially CsPbX_3_ where X = Cl, Br, I) have received massive attention in recent years as favorable optoelectronic materials due to their broad absorption,^1^ intense photoluminescence (PL),^1^ high photoluminescence (PL) quantum yield (QY),^2^ very narrow emission line width,^3^ high defect tolerance,^4^ and wide-range band gaps which can be tuned by controlling the composition^5,6^ and morphology.^7,8^ Due to these interesting properties exhibited by the lead halide perovskite NCs they have been examined in the context of their use in optoelectronic devices, such as photovoltaics,^9,10^ light emitting diodes,^11–15^ lasers^16^ and photodetectors.^17^ In the recent past, numerous other types of cesium lead halide NCs, with structural dimensionality different from the three dimensional orthorhombic/cubic CsPbX_3_ have gained a lot of attention.^18–25^ The structural dimensionality in these structures depends on the extent of coupling of PbX_6_ octahedra in the crystal. If all the corners of the lead hexahalide octahedra are shared, then they are electronically coupled in all three dimensions and referred to as 3D perovskites. If all the corners of the lead hexahalide octahedra are not shared, they are referred to as 0D perovskites.^25^

It is extensively recognized that the excellent optical properties in lead halide based perovskite materials come from the high defect tolerance.^4^ Even the highly luminescent perovskite NCs prepared by the hot injection method are really not defect-free, and these defects serve as trapping centers for exciton recombination and decrease the photoluminescence quantum yields (PLQYs) of the NCs.^26–33^ Different groups have recently synthesized 0D colloidal Cs_4_PbBr_6_ NCs and it has been found that these nanocrystals don't exhibit any photoluminescence.^25^ However, the non-luminescent 0D Cs_4_PbBr_6_ NCs can be converted to 3D CsPbBr_3_ NCs using additives like water,^34^ PbBr_2_ ^35^ ligands,^36^ and Prussian blue.^37^ The non-luminescent nature of Cs_4_PbBr_6_ at room temperature is attributed to the origin of structural and point defects.^38^ Nikl *et al.* observed the true emission of Cs_4_PbBr_6_ at low temperatures (4 K) and the true emission is centered at 375 nm. In the case of thin films at room temperature, Cs_4_PbBr_6_ is mixed with traces of the CsPbBr_3_ phase, which in turn results in the emission at 545 nm. However in the case of nanocrystals, no emission has been observed so far for the pure Cs_4_PbBr_6_ nanocrystals.

Recently it has been shown that addition of appropriate metal halide salts significantly enhances the photoluminescence intensity of CsPbBr_3_ nanocrystals by improving the surface capping. Herein we demonstrate ZnX_2_ mediated post-synthetic transformation of Cs_4_PbBr_6_ nanocrystals (NCs) into CsPbX_3_ nanostructures through an intermediate composite Cs_4_PbX_6_/CsPbX_3_. We find that this procedure results in a highly reproducible increase of the PL QY of up to 90% for the ZnX_2_ treatment. The transformation takes place through the surface passivation of Cs_4_PbBr_6_ nanocrystals with ZnX_2_ followed by anion exchange (in the case of ZnI_2_ and ZnCl_2_) resulting in the formation of an intermediate Cs_4_PbX_6_@CsPbX_3_ passivated by ZnX_2_ followed by the extraction of CsBr from the composite nanostructures. Interestingly, as soon the ZnX_2_ is added to the nanocrystals, excitonic absorption resulting from the Cs_4_PbBr_6_ particles passivated with ZnX_2_ is observed. This is the first time that the true emission of the Cs_4_PbX_6_ crystal structure in the nanocrystalline phase was observed at room temperature.

**Fig. 1:**
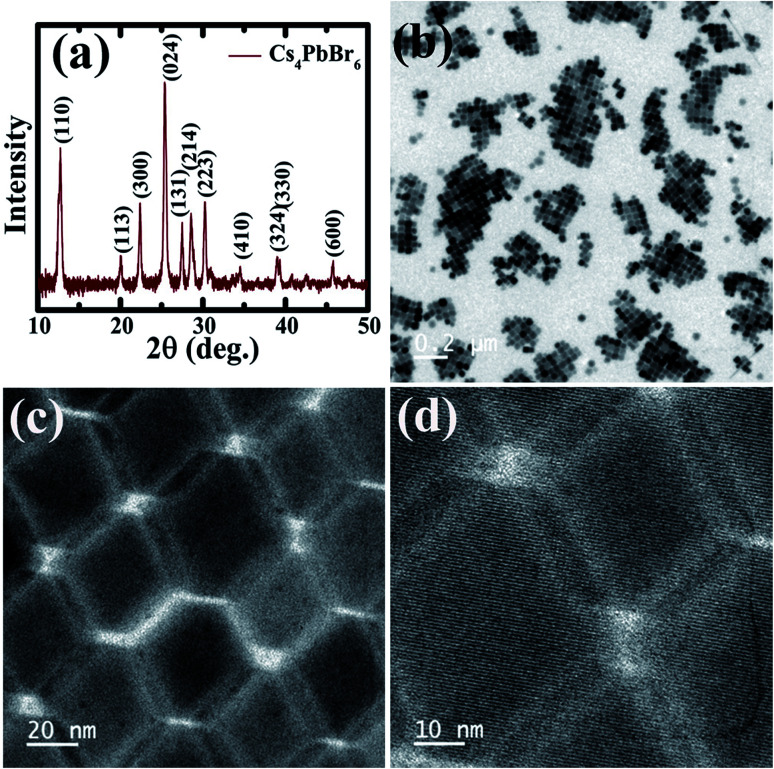
(a) X-ray diffraction patterns obtained of the pristine Cs_4_PbBr_6_ NCs. (b–d) TEM images of the pristine Cs_4_PbBr_6_ NCs at different magnifications.

The Cs_4_PbBr_6_ nanocrystals were synthesized according to the hot injection strategy as described in the Experimental section. The Cs_4_PbBr_6_ nanocrystal dispersion in hexane is completely transparent and as shown in the TEM images in Fig. 1b–d, the pristine Cs_4_PbBr_6_ nanocrystals consist of hexagons of around 30 nm in size. The powder diffraction pattern indicated that the nanocrystals crystallized in the rhombohedral Cs_4_PbBr_6_ phase in the space group *R*3*c*.

**Fig. 2:**
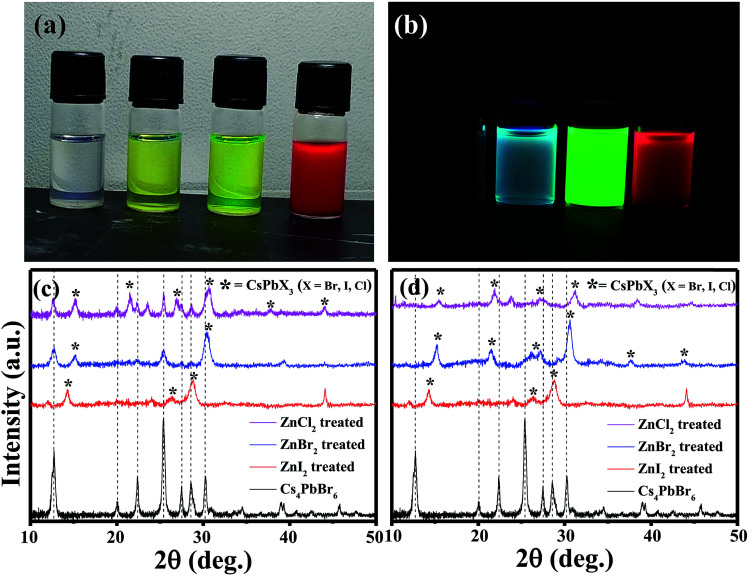
Photographs of the solutions of Cs_4_PbBr_6_, ZnCl_2_ treated, ZnBr_2_ treated and ZnI_2_ treated NCs, respectively under ambient light (a) and under UV light (b). (c) X-ray diffraction patterns obtained for the pristine and ZnX_2_ treated NCs. (d) X-ray diffraction patterns obtained of the samples left without centrifugation after the addition of ZnX_2_.

The pristine Cs_4_PbBr_6_ nanocrystals (NCs) were post treated with a ZnX_2_/hexane solution and used for further characterization. Upon addition of ZnX_2_ (X= Cl, Br, I) solution, clear color change was observed (Fig. 2a). And the initially non-luminescent solution turned into a brightly luminescent solution under UV light after the post-treatment (Fig. 2b). To understand the reason for the observed color changes, powder diffraction analysis was carried out. Fig. 2c shows the X-ray diffraction patterns obtained for the pristine and ZnX_2_ treated NCs. The XRD pattern of the parent NCs indicated that they are crystalline and matched well with the reported rhombohedral Cs_4_PbBr_6_ phase. No characteristic reflections of CsPbBr_3_, CsBr or PbBr_2_ were observed. Upon treating with ZnBr_2_, new reflections were observed along with the reflections from Cs_4_PbBr_6_. All the new reflections could be attributed to the CsPbBr_3_ phase and the post treatment resulted in the formation of the composite Cs_4_PbBr_6_/CsPbBr_3_. Similar behavior was observed in the case of ZnI_2_ treated and ZnCl_2_ treated NCs wherein a composite of Cs_4_PbX_6_/CsPbX_3_ is formed. In the case of ZnI_2_ treated NCs, the pristine Cs_4_PbBr_6_ reflections were shifted towards the lower 2*θ* indicating that anion exchange took place from the bromide to iodide resulting in the formation of Cs_4_PbI_6_. A slight shift towards higher 2*θ* was observed in the case of ZnCl_2_ treated NCs. Interestingly, when the ZnX_2_ post treated solutions were left without centrifugation for a few days, the luminescence of the solution was reduced in comparison to that of the samples which were centrifuged and washed after the ZnX_2_ post-treatment. We have carried out XRD analysis on those samples left without centrifugation and Fig. 2d shows the diffraction patterns obtained for the same samples. From the diffraction patterns it can be seen that the ZnBr_2_ left in the solution results in the complete transformation of 0D Cs_4_PbBr_6_ NCs into 3D CsPbBr_3_ NCs. In the case of ZnI_2_ post-treatment, the reflections from 0D Cs_4_PbI_6_ still exist, indicating that the transformation from 0D to 3D is not complete even after a few days.

**Fig. 3:**
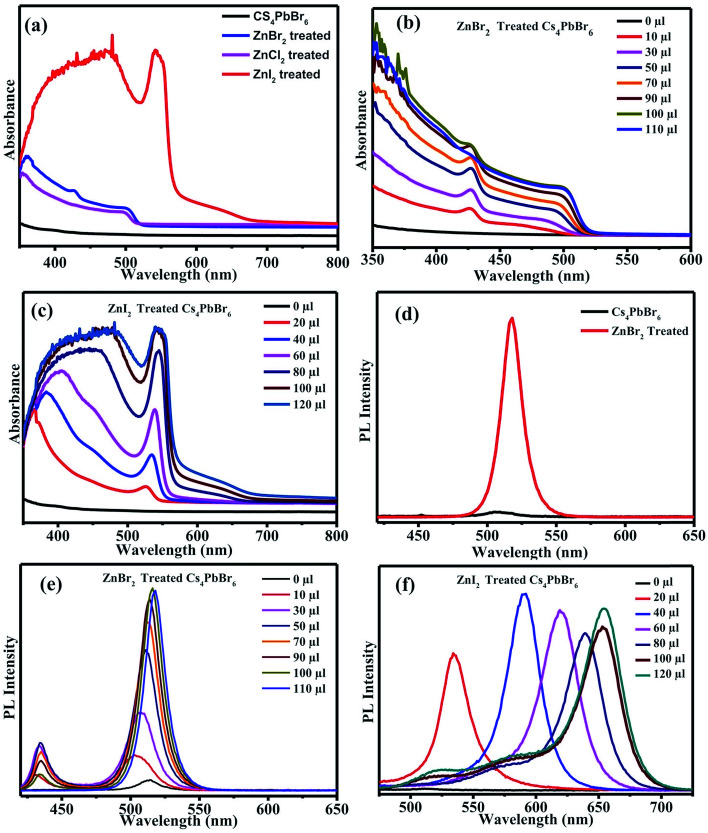
(a) UV-vis absorption spectra of the ZnX_2_ treated Cs_4_PbBr_6_ nanocrystals. (b) Absorption spectra measured using different concentrations of the ZnBr_2_/hexane solution. (c) Absorption spectrum of ZnI_2_ treated NCs with the different concentrations of the ZnI_2_/hexane solution. (d) PL spectra of the pristine NCs and ZnBr_2_ treated NCs. PL spectra of the Cs_4_PbBr_6_ NCs with different concentrations of (e) ZnBr_2_ solution and (f) ZnI_2_ solution.

The absorption spectra of the ZnX_2_ treated Cs_4_PbBr_6_ NCs are shown in Fig. 3a. For the pristine nanocrystals, the absorption and the photoluminescence features are consistent with those in the previously reported studies. The absorption spectra of the as-prepared nanocrystals feature a strong and narrow absorption at 313 nm. The long tail in the absorption spectrum indicates that a certain degree of coupling exists between the PbBr_6_ octahedra. Fig. 2a and b show the photographs of the pristine Cs_4_PbBr_6_ and ZnX_2_ treated NCs under room light and UV light. Upon treating the NCs with the ZnBr_2_/hexane solution, the initially colorless solution turned green color. The same is observed in the UV-vis absorption spectrum with a red shift of the absorption band edge to 510 nm. The features are similar to the characteristic feature of the 3D CsPbBr_3_ NCs,^1–10^ probably indicating the transformation of Cs_4_PbBr_6_ into CsPbBr_3_ NCs. Along with this, another hump was observed at 420 nm in the ZnBr_2_ treated sample. This hump was not observed previously in the pure CsPbBr_3_ NCs,^1–10^ the ZnBr_2_ stabilized CsPbBr_3_ NCs and the Zn^2+^ doped CsPbBr_3_ nanocrystals.^39^ To see if similar behavior is observed with other zinc halides, the Cs_4_PbBr_6_ NCs were also treated with ZnI_2_ and ZnCl_2_. ZnCl_2_ treatment resulted in a slight blue shift in comparison to ZnBr_2_ treated NCs. However, in the case of ZnCl_2_ treated NCs, no hump in the absorption spectrum was evident. In the case of ZnI_2_, an increased red shift (up to 670 nm) in comparison to that in the ZnBr_2_ treatment was observed. Along with the red shifted absorption, a sharp peak at 550 nm can be seen.

To confirm if the peak observed at 420 nm arises from the sample, an aliquot of the Cs_4_PbBr_6_ NCs was taken in a cuvette, different concentrations of ZnBr_2_ were added to the sample and the absorption spectrum of the ZnBr_2_ treated NCs was measured. Fig. 3b shows the absorption spectra measured using different concentrations of the ZnBr_2_/hexane solution added to the cuvette containing NCs. A sharp absorption peak at 425 nm along with a long tail extending up to 510 nm was observed. The absorption intensity of the tail increased gradually and a sharp band edge at 510 nm, which is a characteristic peak of CsPbBr_3_, was observed. The peak at 425 nm is blue shifted to 420 nm and is masked (though visible) by the strong absorption of CsPbBr_3_ in this region. Fig. S1† shows the comparison of the absorption spectra of the CsPbBr_3_ nanocrystals synthesized and the ZnBr_2_ treated Cs_4_PbBr_6_ NCs. A similar concentration dependent study was carried out in the case of ZnI_2_ treated Cs_4_PbBr_6_ NCs. Fig. 3c shows the evolution of the absorption spectrum of ZnI_2_ treated NCs with different amounts of ZnI_2_ added. A sharp absorption at 540 nm is evident even with high amounts of added ZnI_2_. ZnBr_2_ treated NC samples were found to be highly stable even after 2 months of maintaining under 70% RH conditions. Fig. S2† shows photographs under UV light for the ZnBr_2_ treated NC sample and the as-prepared CsPbBr3 NCs after storing under 70% RH conditions.

To corroborate the observed absorption behavior, photoluminescence (PL) studies were also carried out on the parent NCs and ZnX_2_ treated NCs. Fig. 3d shows the PL spectra of the pristine NCs and ZnBr_2_ treated NCs. No strong PL emission was observed, which is consistent with the previously observed decay of excitonic PL above temperatures of 100 K. Very weak PL emission centered at 515 nm was observed which can be attributed to the CsPbBr_3_ phase embedded in the PL-inactive Cs_4_PbBr_6_ crystalline matrix. With the ZnBr_2_ post-treated NC solution, strong PL was observed at 518 nm. From the literature, it is known that the excitonic absorption peak of pure CsPbBr_3_ NCs is located at around 510 nm. So, it is reasonable to speculate that the strong excitonic absorption peak and strong PL peak at 518 nm in Fig. 3d originate from the CsPbBr_3_ NCs, indicating that a transformation might have taken place from Cs_4_PbBr_6_ to CsPbBr_3_.

We also carried out ZnBr_2_ concentration dependent emission studies. With the addition of ZnBr_2_, two emission peaks were observed, one at 430 nm and the other at 510 nm, similar to the behavior observed in the absorption spectrum. The emission at 430 nm could be attributed to the 425 nm peak observed in the absorption spectrum and the peak at 510 nm could be attributed to the band edge emission corresponding to the absorption edge shown in Fig. 3e. Similar behavior was also observed in the case of the iodide counterpart, wherein the two emission peaks corresponds to the absorption peak at 550 nm and band edge absorption (Fig. 3f).

Together with the results obtained from the XRD studies and UV-vis and PL spectra, we speculate that CsPbX_3_/Cs_4_PbX_6_ composites were formed after the post-treatment with ZnX_2_. In the case of ZnBr_2_, the excitonic CsPbBr_3_ absorption was observed at 510 nm and the absorption peak observed at a lower wavelength could be attributed to the Cs_4_PbBr_6_ phase. It has been previously reported that defect states created due to the surface halide vacancies could be responsible for quenching the true emission in Cs_4_PbBr_6_ and were only observed at low temperatures (4 K). When the Zn^2+^ salts were added to the Cs_4_PbBr_6_ NCs, the surface halide defect states could be passivated and the true emission from the surface passivated/modified Cs_4_PbBr_6_ nanocrystals could be observed at 430 nm. The true emission of Cs_4_PbBr_6_ is observed for the first time at room temperature when Zn^2+^ salts are used to passivate the surface defects; however, the Zn^2+^ salts also triggered the transformation of 0D Cs_4_PbBr_6_ NCs into 3D CsPbBr_3_ NCs. So, the intensity of the true emission of Cs_4_PbBr_6_ NCs gradually decreased as the concentration of the zinc salt is increased. When the concentration of the zinc salt was kept constant at 50 μL of ZnBr_2_, the intensity gradually decreased indicating that the whole process takes place through a two-step mechanism. Similar studies carried out using other salts like Cu^2+^ and Mn^2+^ didn't result in the enhancement of the PL QY. In the case of Mn^2+^ a slight increase was observed but Cu^2+^ didn't show any improvement in the PL QY. Fig. S3† shows the PL of the CuBr_2_ and MnBr_2_ treated Cs_4_PbBr_6_ NCs.

**Fig. 4:**
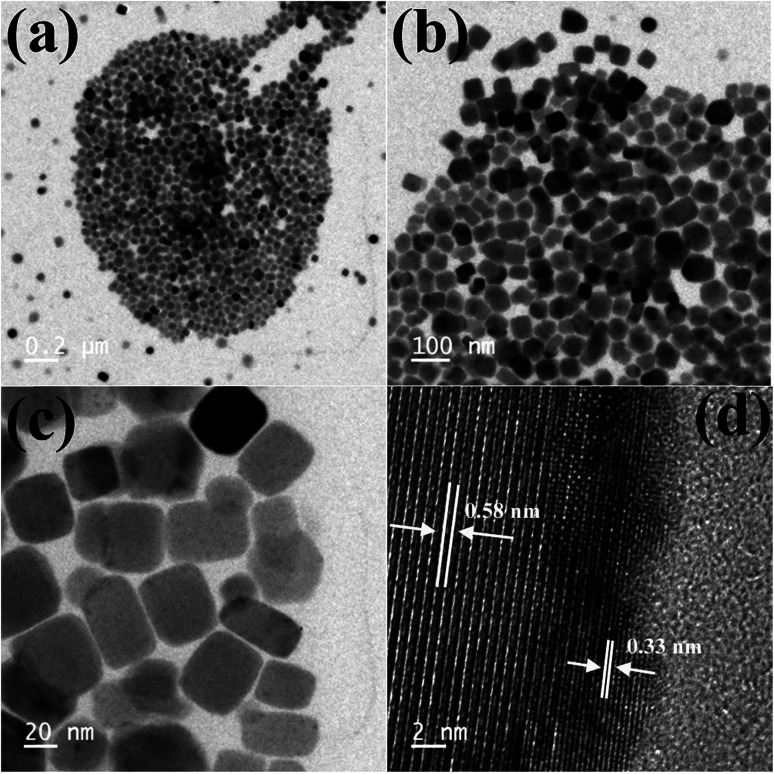
TEM images of the ZnBr_2_ treated Cs_4_PbBr_6_ NC samples (a–c) and HRTEM (d) image of the ZnBr_2_ treated Cs_4_PbBr_6_ NC samples indicating the formation of a composite of CsPbBr_3_/Cs_4_PbBr_6_ NCs.

TEM images of the as-synthesized Cs_4_PbBr_6_ NCs show hexagonal shaped particles of around 30–35 nm in size similar to the particles reported previously. Fig. 4 shows the TEM images of the as-synthesized Cs_4_PbBr_6_ NCs and the ZnBr_2_ treated samples. When the samples were treated with 50 μL of ZnBr_2_, initially hexagonal shaped particles were converted mostly to cubic shaped particles as shown in Fig. 4. More than 90% of the particles became cubic; however the cubic shaped particles did not have sharp edges as observed in the case of the as-prepared CsPbBr_3_ NCs (Fig. S4†). The HR-TEM image (Fig. 4d) shows a lattice spacing of 0.58 nm for the (100) plane of CsPbBr_3_ after ZnBr_2_ treatment. The edges of the particles, however, show that the lattice spacing is not the same as that in the center of the particles. The lattice spacing of 0.33 nm for the (411) plane corresponds to the presence of Cs_4_PbBr_6_, indicating the formation of a composite of CsPbBr_3_/Cs_4_PbBr_6_. A plausible two step formation mechanism can be inferred from the observed TEM images. As the Cs_4_PbBr_6_ nanocrystals were treated with the zinc halide salts, in the first step Zn^2+^ ions could passivate the surface of the nanocrystals and then result in the extraction of excess Cs^+^ ions, and an increase in the dimensionality from 0D to 3D is observed. In the second step we believe that an inside out diffusion of Cs^+^ ions takes place resulting in the formation of CsPbBr_3_/Cs_4_PbBr_6_ composites.

**Fig. 5:**
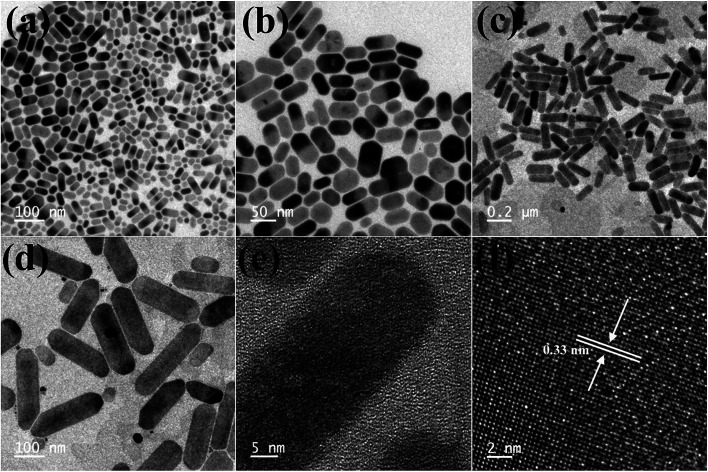
TEM images obtained after the 40 μL ZnI_2_ post treatment (a and b) and 100 μL ZnI_2_ post treatment (c–f). HRTEM images showing the lattice spacing after the post treatment with 100 μL ZnI_2_ (e and f).

When the Cs_4_PbBr_6_ NCs were treated with 40 μL of ZnI_2_ solution, the shape of the particles changed from hexagonal to rod shaped. As seen in the TEM images shown in Fig. 5b the corners of the rods still have hexagonal facets. With the increase in the concentration of the ZnI_2_ solution to 100 μL the length of the rods increased to nearly 250 nm and the width increased to 20–30 nm.

To see if any zinc is present on the surface of the nanocrystals, resulting in the enhanced PL QY, X-ray photoelectron spectroscopy (XPS) measurements have been used to assess the surface modification of the NCs and to understand the mechanism of PL enhancement. After measurements, all core level spectra were calibrated using the C 1s peak at 285.0 eV. The high-resolution XPS spectrum of untreated NCs for Pb 4f yields 2 peaks as shown in Fig. S5.† Two intense peaks are observed at 138 eV (4f_7/2_) and 143 eV (4f_5/2_) with a spin–orbit splitting of nearly 5 eV, which corresponds to Pb in the 2+ oxidation state. Quantitative XPS analysis indicates that Cs : Pb : Br = 1.5 : 1 : 4.2 for untreated Cs_4_PbBr_6_ NCs, which is Pb rich in composition. However, for ZnBr_2_-Cs_4_PbBr_6_ NCs, the composition becomes anion-rich (Cs : Pb : Br = 0.6 : 1 : 5.5), which was also repeatedly observed (Fig. S6†). A small fraction of the Zn^2+^ was also observed in the ZnBr_2_ treated Cs_4_PbBr_6_ NCs, with the ratio of Pb : Zn = 1 : 0.08. This also conforms with the previous observations that the Zn^2+^ ions modify the surface of the nanocrystals, which then results in the extraction of excess Cs^+^ ions from the Cs_4_PbBr_6_ NCs. Furthermore, the Pb/Cs ratio increased from 0.66 to 1.66 in ZnBr_2_ treated Cs_4_PbBr_6_ NCs compared with pristine-Cs_4_PbBr_6_ NCs indicating that ZnBr_2_ extracted excess Cs^+^ ions present. This is also consistent with the XRD analysis wherein the Cs_4_PbBr_6_ is converted to CsPbBr_3_ after the extraction of cesium ions. We have also carried out XPS analysis of the ZnI_2_ treated Cs_4_PbBr_6_ NCs and found that the ratio of Pb : Zn (1 : 0.15) is much higher compared to that in the bromine case (Fig. S7†).

**Fig. 6:**
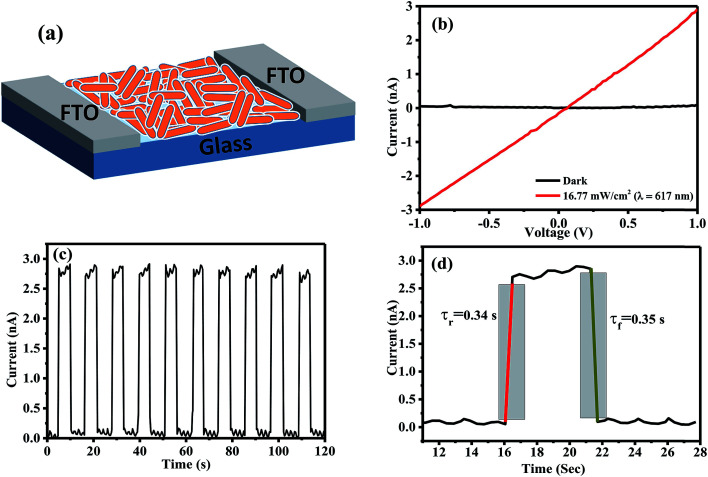
(a) Schematic of the photo-detector architecture. (b) *I*–*V* curves in the dark and in the presence of 617 nm light. (c) Photocurrent time characteristics of the photo detector in the dark and under light (617 nm, 1 volt). (d) Rise and decay time.

Owing to the one-dimensional nature of the nanorods in the case of ZnI_2_ treated Cs_4_PbBr_6_ NCs, we have explored their potential application in optoelectronic devices. To this end, we have fabricated photodetectors by dropcasting the ZnI_2_ treated NCs in hexane between two fluorine doped tin oxide (FTO) electrodes. Fig. 6a shows the schematic of the device architecture that is employed for the fabrication of the devices. The dark current observed was in the order of a few picoamperes, within the detection limit of our instrument. The current of the same device increased under illumination using a diode source of 617 nm, demonstrating their potential as an efficient photodetector. Fig. 6b shows the IV curves obtained in the dark and in the presence of the light diodes. The ratio of the photocurrent to dark current could reach over two orders of magnitude. Time-dependent photocurrent measurements were carried out by pulsing the diode at a fixed light intensity with an applied bias voltage of 2 V. Prompt and reproducible photocurrent response to the pulsing was observed indicating the excellent optical switching and stability of the photodetectors. The rise time and decay time were also determined from the pulsing experiments and they are in the order of a few milliseconds as shown in Fig. 6d. When the photodetectors were fabricated using ZnBr_2_ treated nanocrystals, the currents obtained were lower compared to those using the ZnI_2_ treated samples. Fig. S8† shows the performance of the photodetectors using ZnBr_2_ treated NCs in the same configuration. This indicates that the performance of the photodetectors could be improved if the length of the nanorods could be made longer than the width of the channel used and also by removing the surface capping ligands by appropriate treatments.

In conclusion, we have developed a simple and convenient post synthetic strategy for the transformation of 0D Cs_4_PbBr_6_ into highly luminescent CsPbBr_3_/Cs_4_PbBr_6_ composites using ZnBr_2_ treatment. The transformation with ZnBr_2_ treatment proceeds through a two-step process, where in the first step, zinc doped/modified Cs_4_PbBr_6_ NCs are formed which then results in the conversion to CsPbBr_3_/Cs_4_PbBr_6_ nanocomposites. The composite of CsPbBr_3_/Cs_4_PbBr_6_ NCs is found to be very highly luminescent with a PL QY of 90%. In the case of ZnI_2_ treatment, the post synthetic treatment also resulted in morphological transformation from the hexagonal shaped nanocrystals to nanorods. The length of the rods could be varied depending on the concentration of the nanocrystals and the zinc iodide salt added. In both the iodide and bromide case an intermediate is formed upon treatment with the zinc salt. The intermediate was observed in the time/concentration dependent UV-vis and PL studies, and from the X-ray diffraction analysis, we presume that it is the 0D structure modified/doped by the zinc cations. Photodetectors based on the ZnI_2_ treated Cs_4_PbBr_6_ NCs were fabricated, which exhibit a high on/off ratio with a fast response time. The excellent optoelectronic properties make this treatment versatile for a wide range of functional optoelectronic devices like light emitting diodes and photovoltaic devices.

## Experimental section

### Chemicals

Cesium carbonate (Cs_2_CO_3_, 99.9%), lead(ii) bromide (98%), zinc(ii) iodide (98%), zinc(ii) chloride (99%), oleylamine (OLA, 70%), oleic acid (90%), and 1-octadecene (90%) were purchased from Sigma Aldrich. Zinc(ii) bromide (98%) was purchased from Alfa Aesar.

### Synthesis of cesium oleate

Cesium carbonate (Cs_2_CO_3_, 160 mg), oleic acid (1 mL), and octadecene (16 mL) were added to a three-neck flat bottom flask and kept under vacuum for 30 min at 120 °C. After 30 minutes the temperature was increased to 150 °C and the system was kept under a N_2_ atmosphere until a clear solution of cesium oleate was obtained.

### Synthesis of Cs_4_PbBr_6_ nanoparticles

Octadecene (10 mL), oleylamine (1 mL), oleic acid (1 mL) and lead bromide (0.36 mmol, 73.4 mg) were loaded into a three-neck round bottom flask and degassed for 30 min at 120 °C. The flask was then filled with N_2_ and the temperature was increased to 140 °C. After that, 4.4 mL of hot cesium oleate solution was injected quickly. After 5 s, the reaction mixture was quenched in an ice-water bath.

### Synthesis of CsPbBr_3_ nanoparticles

Lead bromide (207 mg) and octadecene (15 mL) were loaded into a three-neck round bottom flask and degassed for 1 hour at 120 °C. The flask was then filled with N_2_ and oleylamine (1.5 mL) and oleic acid (1.5 mL) were added. After complete solubilisation of PbBr_2_, the temperature was increased to 165 °C and finally the as-prepared Cs-oleate (1.2 mL) was injected. The reaction was immediately quenched in an ice-water bath.

### Purification of nano particles

The crude solution of nanoparticles was first centrifuged at 6000 rpm for 10 min. After that, the supernatant was discarded and the precipitate was redispersed in hexane solution. Then again, the large particles present in the solution were removed by centrifugation at 6000 rpm for 10 min. The supernatant was discarded and a colloidal solution of nanoparticles was obtained in hexane.

### ZnX_2_ (X = Br, I and Cl) treatment

The NCs dispersed in hexane (∼8 mg mL^−1^) were treated with the pre-made zinc halide solution. Zinc halide solutions were prepared by dissolving zinc halides (ZnX_2_, X= I, Br, Cl) (0.05 M) in the mixture of hexane (2 mL) and oleylamine (40 μL) at room temperature. Then the required zinc halide solution was added to 1 mL of the Cs_4_PbBr_6_ NCs in hexane solution (8 mg mL^−1^) under constant stirring. Excess zinc halide salts are removed by centrifugation and the NCs can be stored in organic solvents like hexane, toluene, *etc.* for a few months.

### Characterization

High resolution transmission electron microscope images were obtained using a Tecnai G2 F30 300 kV transmission electron microscope. X-ray diffraction spectra were obtained by using a Rigaku Japan SmartLab X-ray diffractometer (Cu Kα, *λ* = 1.541). PL spectra were collected by using a Horiba FluoroMax spectrometer with 400 nm excitation wavelength, while UV-vis absorption measurement was performed using a UV-vis-NIR spectrometer (PerkinElmer LAMBDA 950). The room-temperature PLQY was calculated according to the following equation^38^
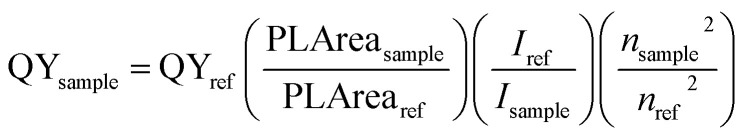


In this equation, QY_sample_ is the PLQY of the sample. PLArea_sample_ is the integrated emission spectrum curve area for the sample. *I*_sample_ is the absorption value at the excitation wavelength for the sample. *n*_sample_ is the refractive index of the employed solvent. QY_ref_, PLArea_ref_, *I*_ref_ and *n*_ref_ are those for the standard reference respectively. In our experiment, fluorescein sodium salt in water and Rhodamine 6G in water (PLQY = 0.95) were chosen to be the standards for green and red spectral regions and the standard solutions should be freshly prepared. Attention was paid to keeping the optical densities of the sample and standard below 0.1 at the excitation wavelength.

### Photodetector fabrication

Laser patterned FTO coated glass with an etched area was used as the photodetector. Before deposition, the laser patterned FTO was cleaned thoroughly by using a soap solution, DI water, acetone, and ethanol for 10 minutes, respectively, by ultra-sonication. Perovskite nanocrystals in hexane were drop cast on the etched area of the FTO coated glass by masking the unetched area with scotch tape. The drop cast substrate was then subjected to vacuum drying. Then the photoresponse was measured under a 617 nm LED light.

## Conflicts of interest

There are no conflicts to declare.

## Supplementary Material

NA-001-C9NA00244H-s001
