# Supplementary material for: ZnX2 mediated post-synthetic transformation of zero dimensional Cs4PbBr6 nanocrystals for opto-electronic applications
Source: Nanoscale Adv. 2019 May 8;1(7):2502–9. doi: 10.1039/c9na00244h (PMC9418536; doi:10.1039/c9na00244h)
Supplement: NA-001-C9NA00244H-s001 [file NA-001-C9NA00244H-s001.pdf]

Electronic Supplementary Material (ESI) for Nanoscale Advances.
This journal is © The Royal Society of Chemistry 2019

Supporting Information

ZnX_2 Mediated Post-Synthetic Transformation of Zero Dimensional Cs_4PbBr_6 Nanocrystals for opto-electronic applications

Sumit Kumar Sharma,[^#] *Swati Mamgain,*[$#] *Burhanuddin Attarwala,*[$] *Aswani Yella*[$*]

^ *Centre of Research in Nanotechnology and Science, Indian Institute of Technology Bombay, 400076, India.*

$Department of Metallurgical Engineering and Materials Science, Indian Institute of Technology Bombay, 400076, India.

# Both the authors contributed equally

** Corresponding Author*

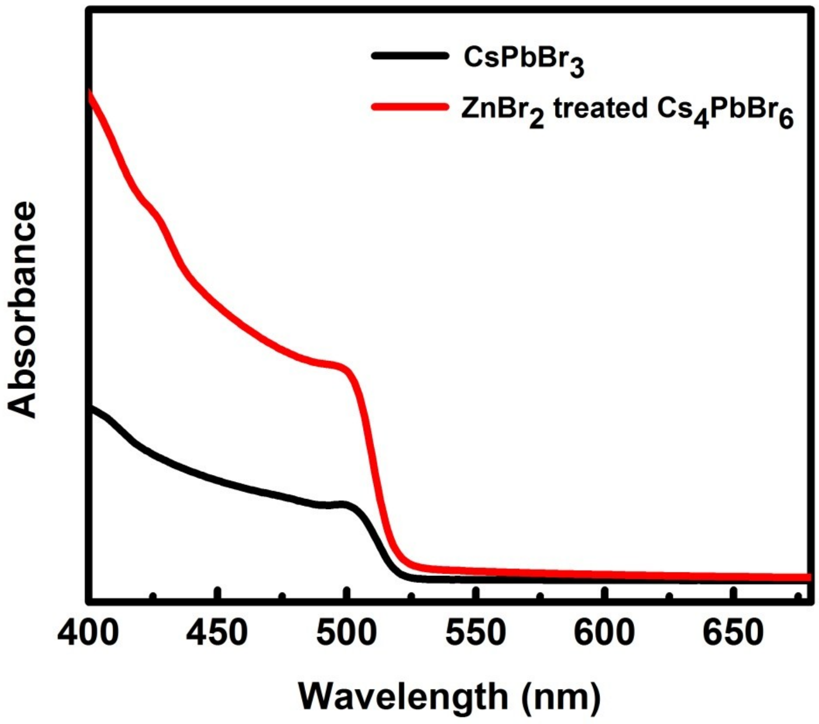

Figure S1: Comparison of the absorption spectra of the $CsPbBr_3$ NCs and $ZnBr_2$ treated Cs_4PbBr_6 NCs.

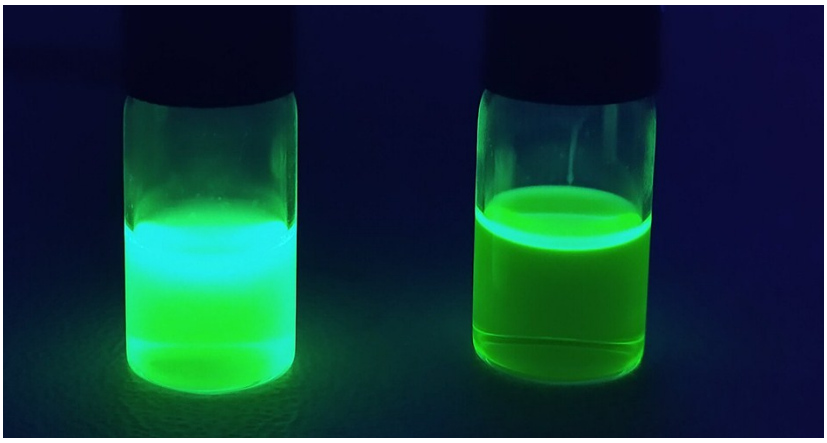

Figure S2. Photograph of the $ZnBr_2$ treated nanocrystal dispersion(left) and $CsPbBr_3$ nanocrystal dispersion (right) after 2 months.

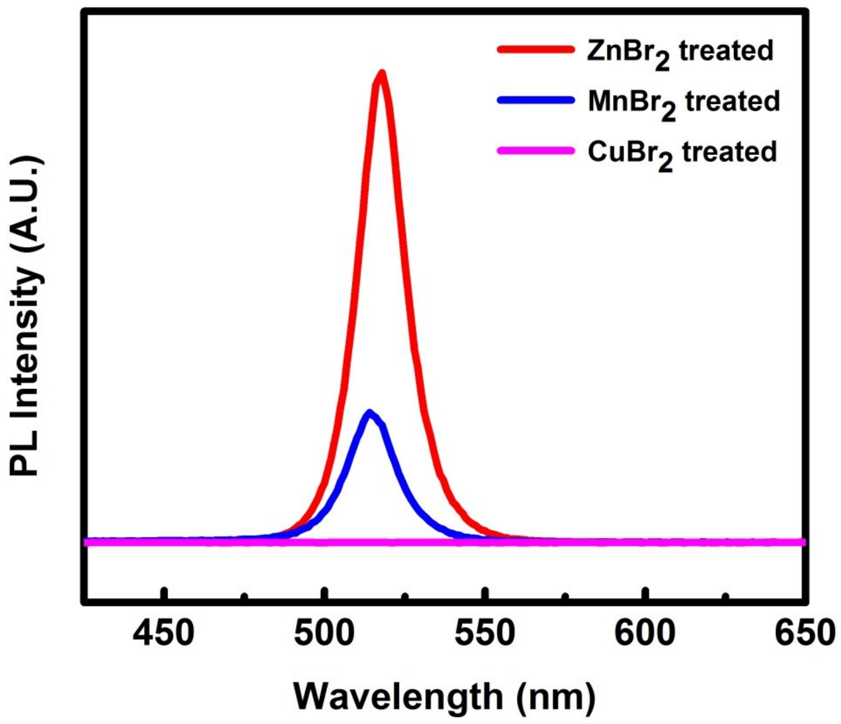

Figure S3: PL of $ZnBr_2$, $MnBr_2$ and $CuBr_2$ treated Cs_4PbBr_6 NCs.

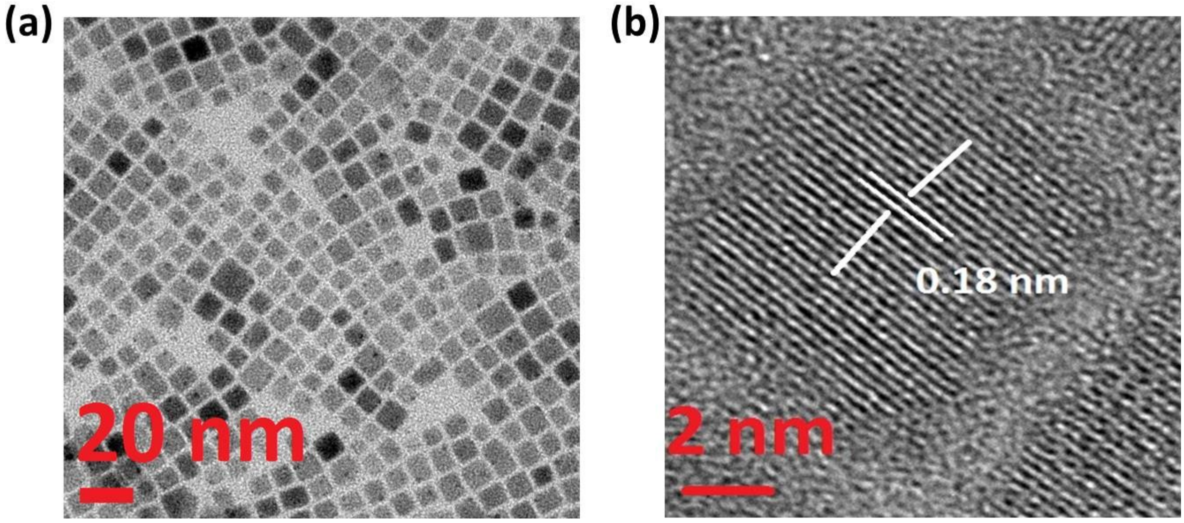

Figure S4: TEM and HRTEM images of $CsPbBr_3$ NCs synthesized by hot injection method.

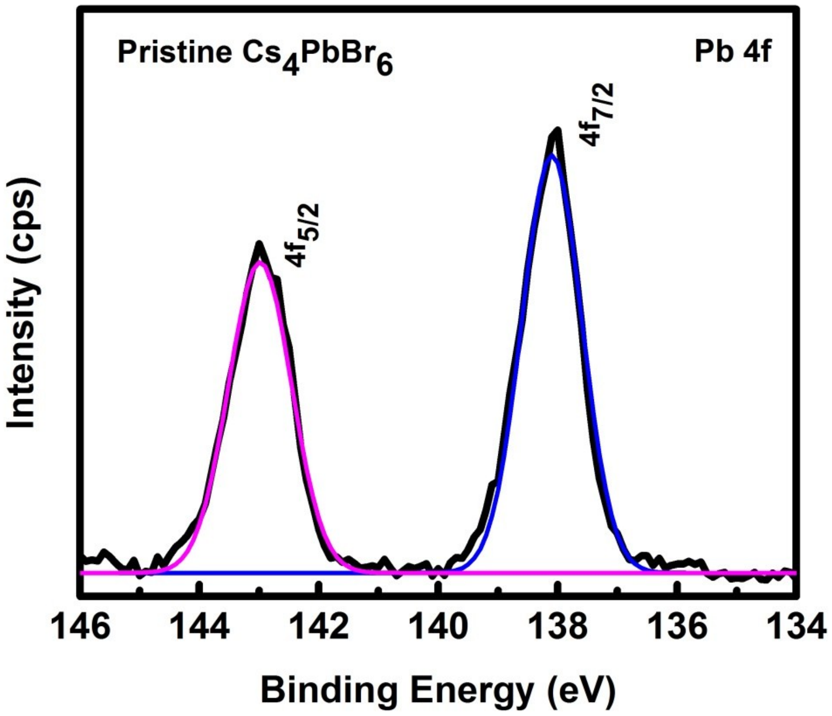

Figure S5: High-resolution XPS spectrum of pristine Cs_4PbBr_6 NCs for Pb 4f.

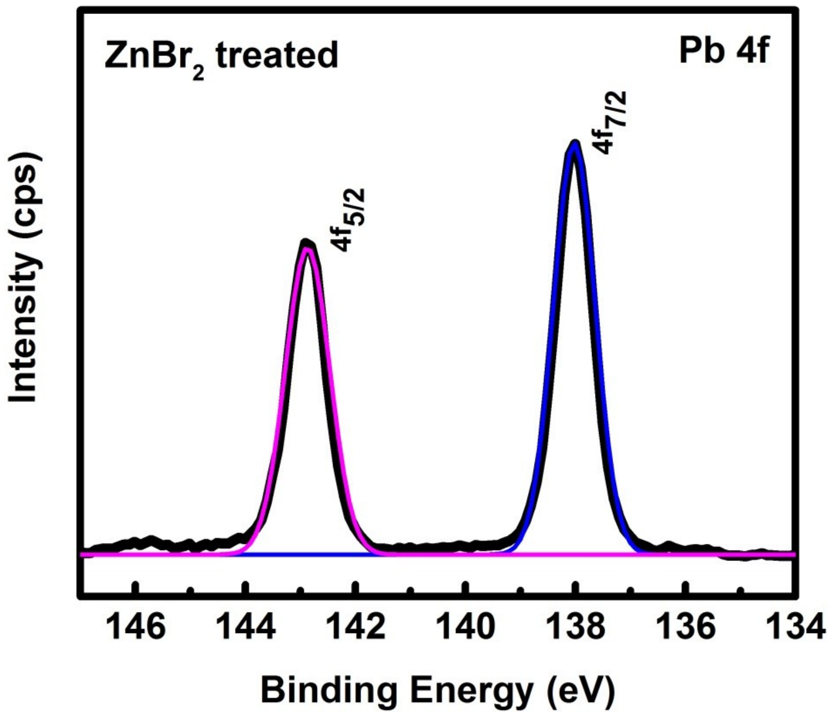

Figure S6: High-resolution XPS spectrum of $ZnBr_2$ treated Cs_4PbBr_6 NCs for Pb 4f.

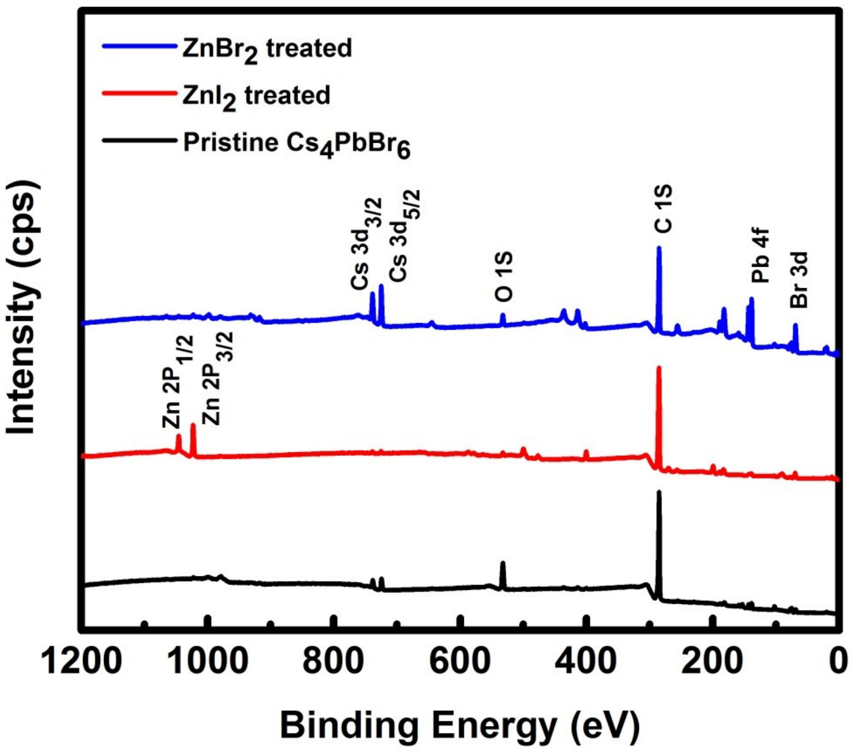

Figure S7: XPS spectra of pristine, $ZnBr_2$ and ZnI_2 treated Cs_4PbBr_6 NCs

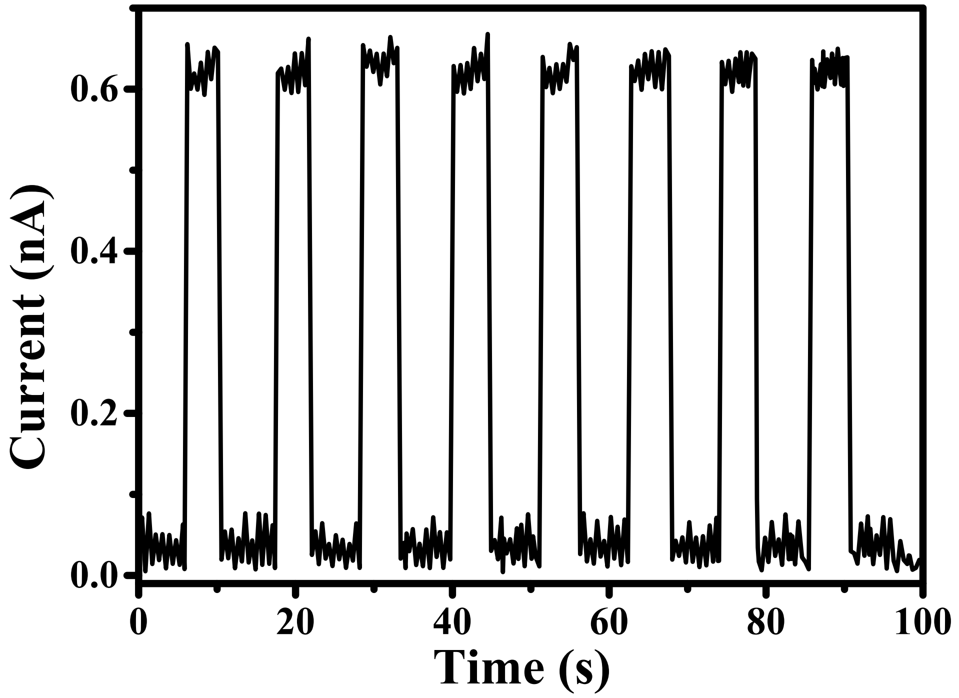

Figure S8 Photodetector response for the device fabricated using $ZnBr_2$ treated NC samples.
